# Pembrolizumab‐Induced Morphea in a Patient With Metastatic Urothelial Carcinoma

**DOI:** 10.1155/criu/2930700

**Published:** 2026-07-22

**Authors:** Matthew A. Drescher, Douglas W. Murray, Douglas J. Grider

**Affiliations:** ^1^ Virginia Tech Carilion School of Medicine, Virginia Polytechnic Institute and State University, Roanoke, Virginia, USA, vt.edu; ^2^ Division of Dermatology, Department of Internal Medicine, Virginia Tech Carilion School of Medicine, Virginia Polytechnic Institute and State University, Roanoke, Virginia, USA, vt.edu; ^3^ Dominion Pathology Associates, Roanoke, Virginia, USA; ^4^ Department of Basic Science Education, Virginia Tech Carilion School of Medicine, Virginia Polytechnic Institute and State University, Roanoke, Virginia, USA, vt.edu

**Keywords:** checkpoint inhibitor, immune-related adverse event, morphea, pembrolizumab, scleroderma, urothelial carcinoma

## Abstract

**Introduction:**

Immune checkpoint inhibitors, such as pembrolizumab, are increasingly used in high‐grade urothelial carcinomas. Immune checkpoint inhibitors are known to cause immune‐related adverse events (irAEs), often involving the skin.

**Case Presentation:**

Reported is a 59‐year‐old woman with metastatic high‐grade urothelial carcinoma of the right renal pelvis previously treated with chemoradiation and surgery. She developed morphea 1.5 years after initiating pembrolizumab therapy. The patient presented with violaceous, indurated plaques and paresthesias of the lower extremities. Biopsies revealed thickened collagen bundles and perivascular inflammation. MRI excluded eosinophilic fasciitis. She was diagnosed with pembrolizumab‐induced morphea and treated with methotrexate and topical corticosteroids, leading to partial improvement.

**Conclusion:**

As immunotherapy becomes more widely used in urologic oncology, recognition of rare cutaneous irAEs such as morphea is crucial for timely diagnosis and management.

Keynote Message

Morphea and scleroderma‐like reactions, though rare, can occur with pembrolizumab treatment for urothelial carcinoma. Awareness of this potential irAE enables earlier recognition and improved patient outcomes.

## 1. Introduction

Immune checkpoint inhibitors (ICIs) have become increasingly important in the treatment of advanced urothelial carcinoma, particularly in patients with recurrent or metastatic disease. Programmed cell death protein 1 (PD‐1) inhibitors such as pembrolizumab improve antitumor immune activity and have demonstrated survival benefit in advanced urothelial carcinoma [1, 2]. However, enhanced immune activation may also result in immune‐related adverse events (irAEs), which frequently involve the skin.

Cutaneous irAEs associated with ICIs range from mild inflammatory eruptions to more severe autoimmune and fibrosing disorders [3, 4]. Although morphea and scleroderma‐like reactions are uncommon, increasing numbers of cases have been reported in association with PD‐1 inhibitor therapy [5, 6]. Recognition of these rare reactions is important, as delayed diagnosis may contribute to progressive fibrosis and functional impairment.

Presented here is a case of pembrolizumab‐associated morphea arising during treatment for metastatic urothelial carcinoma of the right renal pelvis.

## 2. Case Presentation

A 59‐year‐old Caucasian woman was diagnosed with right‐sided malignant urothelial carcinoma of the renal pelvis. Histopathology from a CT‐guided biopsy showed a poorly differentiated carcinoma that was cytokeratin 7 positive and p63 strongly positive without frank metastasis, interpreted as high‐grade urothelial carcinoma. Initial staging was T4N0M0.

Following three cycles of cisplatin and gemcitabine, an MRI of her abdomen showed a slight decrease in tumor size with increased central necrosis. The MRI also demonstrated tumor involvement of the right kidney, right psoas muscle, 10th rib, and possibly the liver. The patient ultimately underwent a right radical nephroureterectomy with liver and diaphragm resection 7 weeks after completing chemotherapy. She was discharged on postoperative day seven.

Surveillance cystoscopy 10 months postoperatively revealed a normal urethra and bladder. However, MRI at 1.5 years postsurgery showed a 3.2‐cm hyperenhancing pleural lesion on the right side, and chest CT revealed a right lung nodule consistent with metastatic recurrence (T4N0M1). Surgical pathology confirmed recurrent urothelial carcinoma. The patient received adjuvant carboplatin and paclitaxel, followed by palliative radiation upon subsequent recurrence 6 years after initial diagnosis. Pembrolizumab therapy was then initiated. A summary of the patient′s oncologic and dermatologic clinical course is provided in Table 1.

**Table 1:** Clinical timeline.

Timepoint	Clinical event
Initial presentation	Diagnosed with high‐grade urothelial carcinoma of the right renal pelvis (T4N0M0) based on CT‐guided biopsy showing poorly differentiated CK7‐positive, p63‐positive carcinoma
Shortly after diagnosis	Received three cycles of cisplatin and gemcitabine
Postchemotherapy imaging	MRI demonstrated slight decrease in tumor size with increased central necrosis; involvement of right kidney, psoas muscle, 10th rib, and possible liver involvement noted
7 weeks after chemotherapy completion	Underwent right radical nephroureterectomy with liver and diaphragm resection
10 months postoperatively	Surveillance cystoscopy showed normal urethra and bladder
1.5 years after surgery	MRI revealed 3.2‐cm right pleural lesion; chest CT demonstrated right lung nodule consistent with metastatic recurrence (T4N0M1)
Following recurrence	Surgical pathology confirmed recurrent urothelial carcinoma
Subsequent treatment course	Received carboplatin and paclitaxel followed by palliative radiation for recurrent disease
6 years after initial diagnosis	Pembrolizumab therapy initiated
Approximately 1.5 years after pembrolizumab initiation	Developed shiny, indurated rash involving legs and abdomen with associated numbness and tingling of lower extremities
Dermatologic evaluation	Physical examination demonstrated violaceous indurated plaques and sclerotic lesions; punch biopsies obtained
Diagnostic workup	Histopathology demonstrated thickened collagen bundles, adnexal entrapment, and mild perivascular infiltrate with eosinophils; MRI excluded eosinophilic fasciitis
Final diagnosis	Pembrolizumab‐induced morphea diagnosed
Treatment for morphea	Treated with methotrexate and topical corticosteroids
Follow‐up	Partial improvement in truncal lesions and mobility after 3 months; methotrexate discontinued after 8 months due to gastrointestinal intolerance with stable residual disease thereafter

Approximately 1.5 years into pembrolizumab therapy, the patient developed a new shiny, tight‐feeling rash on her legs and abdomen with concurrent numbness and tingling in her legs. Gabapentin provided mild relief. She denied dysphagia, dyspnea, Raynaud′s phenomenon, or mouth stiffness.

**Figure 1:**
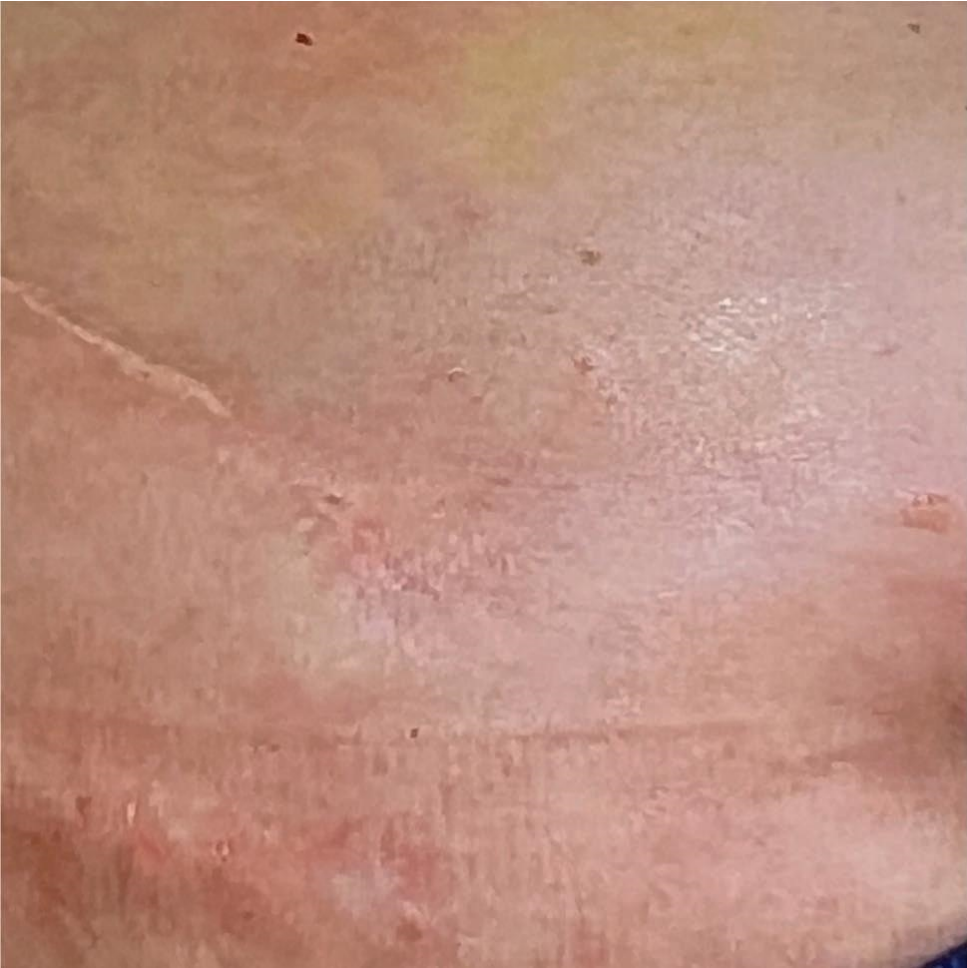
Close‐up photograph of right flank with prior surgical site and indurated plaque with erythema.

**Figure 2:**
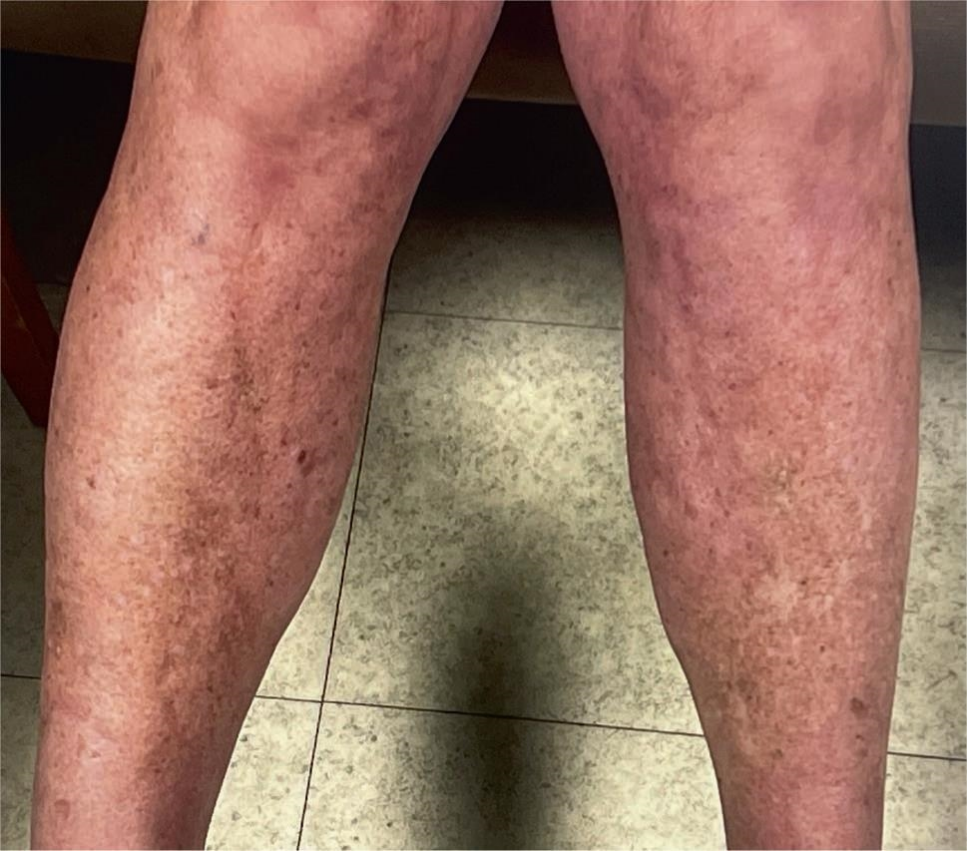
Clinical photograph of the bilateral anterior lower extremities demonstrating firm sclerotic plaques consistent with morphea‐associated cutaneous fibrosis.

**Figure 3:**
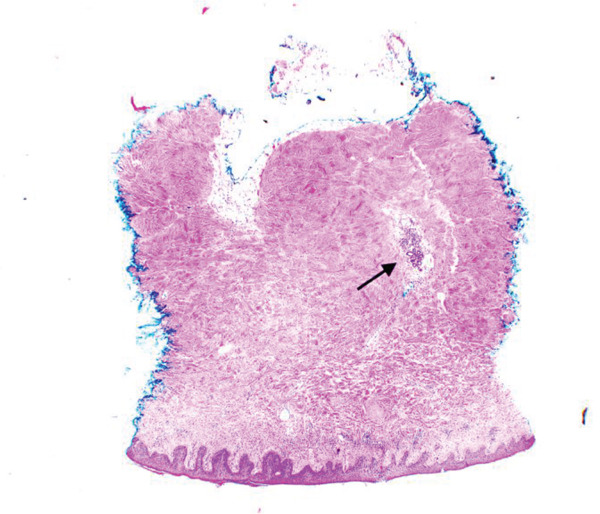
Punch biopsy of the right flank demonstrating thickened reticular dermal collagen with adnexal entrapment involving eccrine structures, consistent with dermal sclerosis (H&E, 2× magnification).

**Figure 4:**
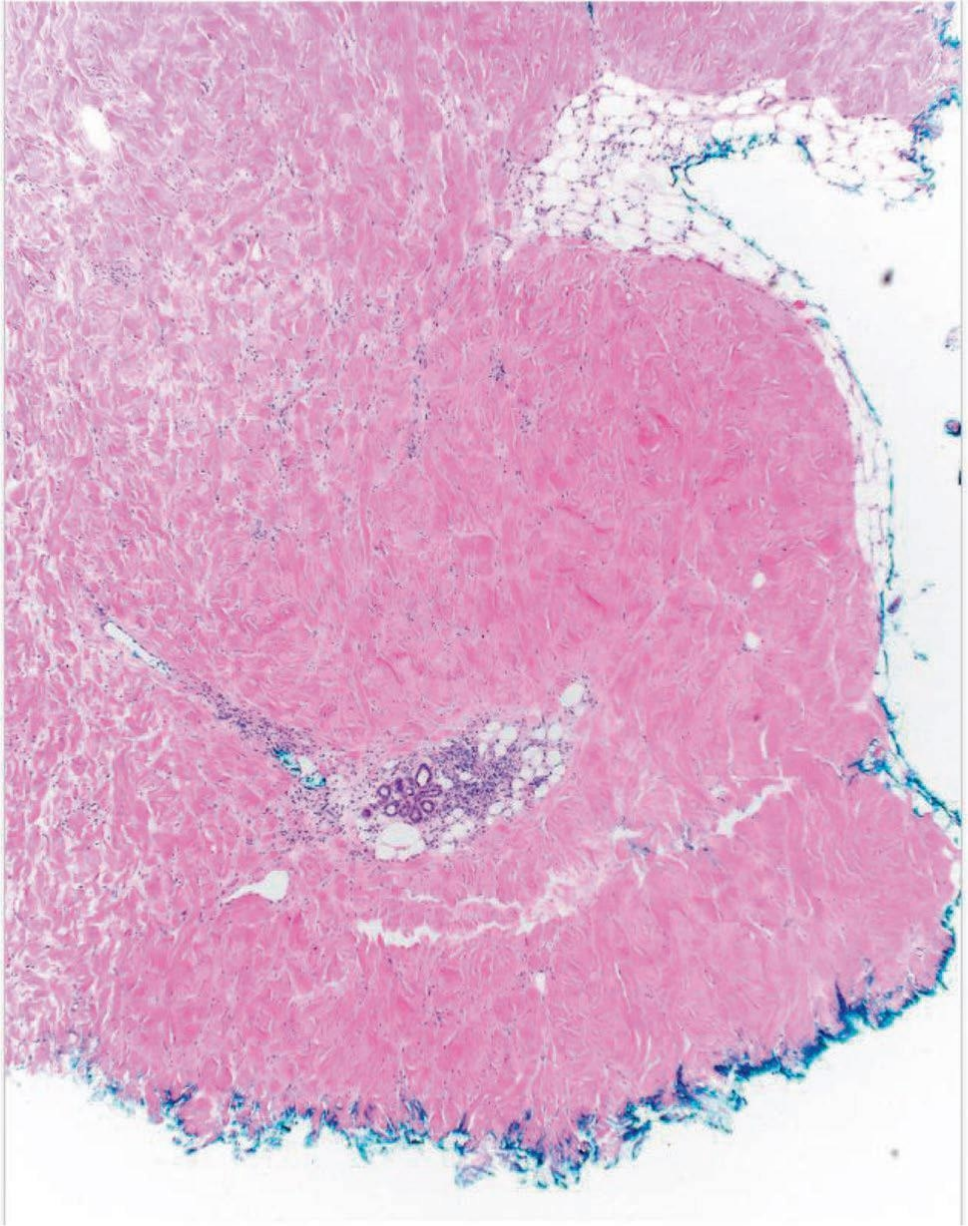
Higher‐power view of the right flank biopsy demonstrating thickened reticular dermal collagen with “bound‐down” eccrine glands, a characteristic feature of morphea (H&E, 4× magnification).

**Figure 5:**
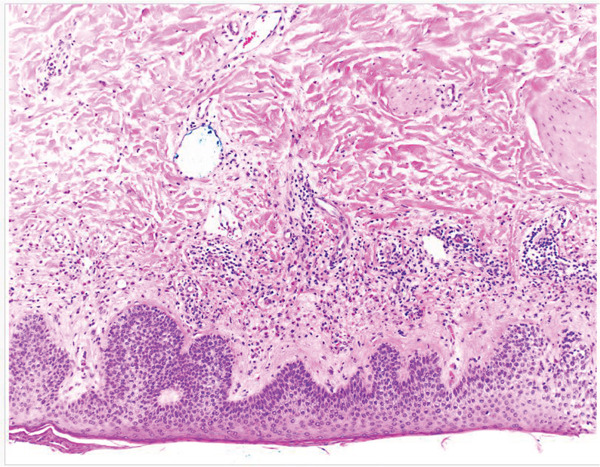
Right flank biopsy demonstrating mild epidermal spongiosis and upper dermal inflammatory infiltrate composed predominantly of lymphocytes with scattered eosinophils, compatible with inflammatory‐stage morphea (H&E, 10× magnification).

Physical examination revealed violaceous, indurated plaques with erythematous borders on the right flank and abdomen (Figure 1) and firm sclerotic plaques on the legs (Figure 2). There was no hand or foot involvement, and nail fold capillaroscopy was normal. Punch biopsies of the right torso and left abdomen both revealed thickened collagen bundles, adnexal entrapment, and mild perivascular infiltrate with scattered upper dermal eosinophils (Figures 3, 4, and 5). MRI ruled out eosinophilic fasciitis, and the diagnosis of pembrolizumab‐induced morphea was made.

The patient was treated with methotrexate and continued topical corticosteroids. Truncal lesions improved within 3 months, with increased walking distance and decreased tightness. Improvement plateaued, and IVIG was recommended but declined. Methotrexate was discontinued after 8 months due to gastrointestinal upset, with stable residual lesions thereafter.

## 3. Discussion

Metastatic urothelial carcinoma has a poor prognosis but has improved with the introduction of immunotherapy. In the Phase III KEYNOTE‐045 trial, pembrolizumab as second‐line therapy improved overall survival compared with chemotherapy alone (median OS 10 vs. 7 months; 44% vs. 30% 1‐year survival) [2]. Pembrolizumab was FDA‐approved in 2017 for locally advanced or metastatic urothelial carcinoma in patients progressing during or within 12 months of platinum therapy, or those ineligible for platinum regimens. Given the rarity of checkpoint inhibitor‐associated morphea and the broad differential diagnosis of sclerosing dermatoses in oncology patients, careful clinicopathologic evaluation was necessary in this case.

As summarized in Table 2, most reported cases of checkpoint inhibitor‐associated morphea and scleroderma‐like reactions have occurred in patients treated for melanoma, with variable timing of onset and generally partial response to immunosuppressive therapy. The differential diagnosis in this case included eosinophilic fasciitis, systemic sclerosis, paraneoplastic scleroderma, radiation‐induced fibrosis, and other drug‐induced scleroderma‐like disorders. Eosinophilic fasciitis is an important consideration in patients receiving ICIs who develop skin induration and extremity tightness, as checkpoint inhibitor‐associated eosinophilic fasciitis has been increasingly reported [10]. Unlike eosinophilic fasciitis, however, this patient demonstrated no MRI evidence of fascial inflammation or thickening. Histopathology additionally favored morphea, demonstrating thickened collagen bundles with adnexal entrapment and mild perivascular inflammation rather than predominant fascial involvement. Although scattered eosinophils were present on biopsy, this finding may also occur in inflammatory‐stage morphea and was not considered sufficient to support eosinophilic fasciitis in the absence of supportive imaging findings.

**Table 2:** Previously reported cases of immune checkpoint inhibitor‐associated morphea and scleroderma‐like reactions.

Author	Malignancy	ICI therapy	Time to onset	Clinical features	Histopathology	Treatment	Outcome
Barbosa et al. [5]	Melanoma	Pembrolizumab	Variable	Diffuse and limited scleroderma‐like changes	Dermal sclerosis with adnexal trapping	Immunosuppression	Partial improvement
Shenoy et al. [7]	Melanoma	Pembrolizumab	Not specified	Severe sclerodermoid reaction	Sclerodermoid dermal fibrosis	Corticosteroids	Persistent disease
Langan et al. [6]	Metastatic melanoma	Anti − PD1 + anti − CTLA4	Delayed	Generalized morphea with indurated waxy plaques	Consistent with morphea	IV dexamethasone, topical steroids, physiotherapy	Partial improvement
Zafar et al. [8]	Melanoma	Pembrolizumab	Variable	Morphea following PD‐1 inhibition	Morphea‐like fibrosis	Immunosuppressive therapy	Partial response
Pai et al. [9]	Breast cancer	Pembrolizumab + chemotherapy	Delayed	Scleroderma‐like plaques/morphea	Compatible with morphea	Corticosteroids and immunosuppressive therapy	Clinical improvement
Present case	Metastatic urothelial carcinoma	Pembrolizumab	~1.5 years	Violaceous indurated plaques with paresthesias	Thickened collagen bundles, adnexal entrapment, and eosinophils	Methotrexate and topical corticosteroids	Partial improvement

Systemic sclerosis was also considered given the patient′s progressive skin induration and sclerosis‐like cutaneous findings. However, several clinical features argued against systemic sclerosis. The patient denied Raynaud phenomenon, dysphagia, dyspnea, and oral tightening, and physical examination demonstrated no sclerodactyly, hand involvement, or nailfold capillary abnormalities. Furthermore, the distribution of disease was localized primarily to the trunk and lower extremities rather than exhibiting the more characteristic acral‐predominant pattern seen in systemic sclerosis. Histopathologic findings additionally favored morphea, as morphea and systemic sclerosis may share overlapping dermal sclerosis but differ substantially in systemic manifestations and vascular features.

Paraneoplastic scleroderma and radiation‐induced fibrosis were also considered. Paraneoplastic scleroderma‐like syndromes have been reported in association with various malignancies and may occasionally precede or accompany cancer recurrence [11]. However, the patient′s dermatologic findings developed after prolonged pembrolizumab exposure in the setting of already established metastatic disease, making a treatment‐related immune phenomenon more likely. Radiation‐induced morphea and fibrosis were also considered given the patient′s prior radiation therapy. Nevertheless, the distribution of the lesions extended beyond previously irradiated fields, arguing against a localized radiation‐induced process.

Although definitive attribution remains challenging in isolated case reports, several features supported pembrolizumab‐associated morphea in this patient. ICIs, particularly PD‐1 inhibitors, have been increasingly associated with scleroderma‐like and morpheaform irAEs in the literature [5, 9, 12]. In the present case, the temporal association with pembrolizumab exposure, compatible clinicopathologic findings, exclusion of major competing diagnoses, and partial response to immunosuppressive therapy collectively supported a treatment‐related immune‐mediated process. Nevertheless, the delayed onset approximately 1.5 years after pembrolizumab initiation and the absence of rechallenge data limit definitive causal inference.

To provide a more structured assessment of causality, the Naranjo Adverse Drug Reaction Probability Scale was applied. The patient received a score of 6, corresponding to a “probable” adverse drug reaction. Factors supporting this assessment included prior published reports of pembrolizumab‐associated morphea and scleroderma‐like reactions, the temporal relationship between pembrolizumab exposure and symptom onset, objective clinicopathologic findings, and exclusion of alternative diagnoses. Nevertheless, the delayed onset of symptoms and lack of rechallenge data preclude definitive attribution.

The delayed onset of cutaneous sclerosis approximately 1.5 years after pembrolizumab initiation is notable but not unprecedented among irAEs associated with checkpoint inhibitors. Although many dermatologic toxicities occur early in treatment, delayed immune‐mediated toxicities have increasingly been recognized, potentially reflecting prolonged immune dysregulation, persistent T‐cell activation, cytokine imbalance, and progressive fibroblast stimulation following PD‐1 pathway inhibition [13–15]. Proposed mechanisms underlying checkpoint inhibitor‐associated morphea include enhanced autoreactive T‐cell activity and increased profibrotic cytokine signaling, including transforming growth factor‐*β*–mediated pathways. The variability in onset timing reported among published cases further suggests that these reactions may emerge after extended immune system modulation rather than immediate hypersensitivity alone.

Management of checkpoint inhibitor‐associated morphea remains largely guided by case reports and broader principles for treatment of immune‐related cutaneous adverse events. Reported therapies have included topical corticosteroids, systemic corticosteroids, methotrexate, mycophenolate mofetil, phototherapy, intravenous immunoglobulin, and other immunosuppressive agents depending on disease severity and functional impairment [6]. In many reported cases, partial improvement rather than complete resolution has been observed, particularly in patients with established fibrotic disease [5]. Early recognition may therefore be important in limiting long‐term sclerosis and mobility impairment. Decisions regarding continuation or discontinuation of ICI therapy should be individualized based on oncologic status and severity of toxicity. In the present case, methotrexate and topical corticosteroids resulted in partial symptomatic and functional improvement, although residual stable sclerosis persisted.

Given pembrolizumab′s growing use, clinicians must remain alert to cutaneous irAEs. Early recognition and immunosuppressive therapy can improve outcomes and preserve function.

## 4. Conclusion

ICIs such as pembrolizumab are effective in treating metastatic urothelial carcinoma. As their use expands in urologic oncology, clinicians should recognize rare irAEs such as morphea to enable timely diagnosis and intervention.

## Author Contributions

Matthew A. Drescher contributed to manuscript drafting, literature review, and manuscript revision. Douglas W. Murray contributed to clinical management, dermatologic evaluation, and manuscript revision. Douglas J. Grider contributed to histopathologic interpretation, manuscript revision, and overall supervision of the project.

## Funding

No funding was received for this manuscript.

## Disclosure

All authors have read and approved the final version of the manuscript. Douglas J. Grider had full access to all data in this study and takes complete responsibility for the integrity of the data and the accuracy of the data analysis.

## Ethics Statement

Written informed consent for publication of this case report and accompanying images was obtained from the patient. This case report describes clinical care performed as part of routine medical practice.

## Conflicts of Interest

The authors declare no conflicts of interest.

## Data Availability

The data that support the findings of this study are available from the corresponding author upon reasonable request.
